# Supplementary material for: ATP increases murine neuroblastoma cell size through a PANX1- and macropinocytosis-dependent mechanism
Source: Biol Open. 2025 Dec 8;14(12):bio062272. doi: 10.1242/bio.062272 (PMC12746712; doi:10.1242/bio.062272)
Supplement: Supplementary information [file biolopen-14-062272-s1.pdf]

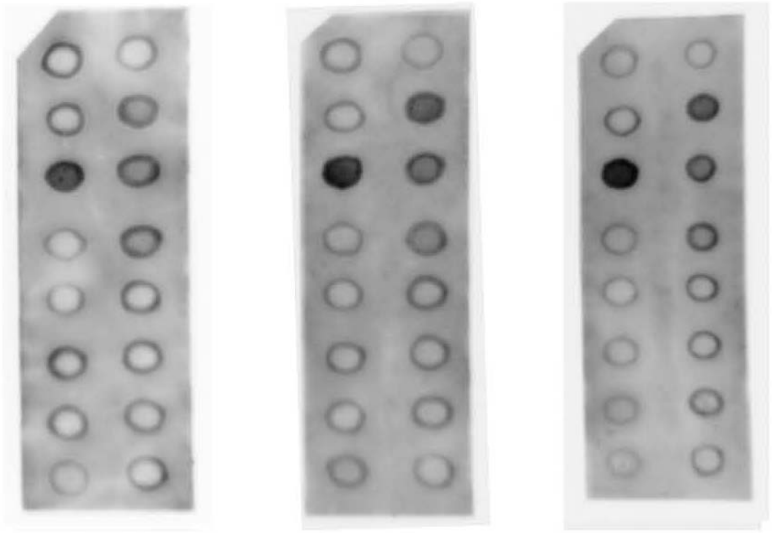

Fig. S1. Full Membrane Lipid Strip blots for Fig. 3 analysis (n = 3)

Biology Open • Supplementary information

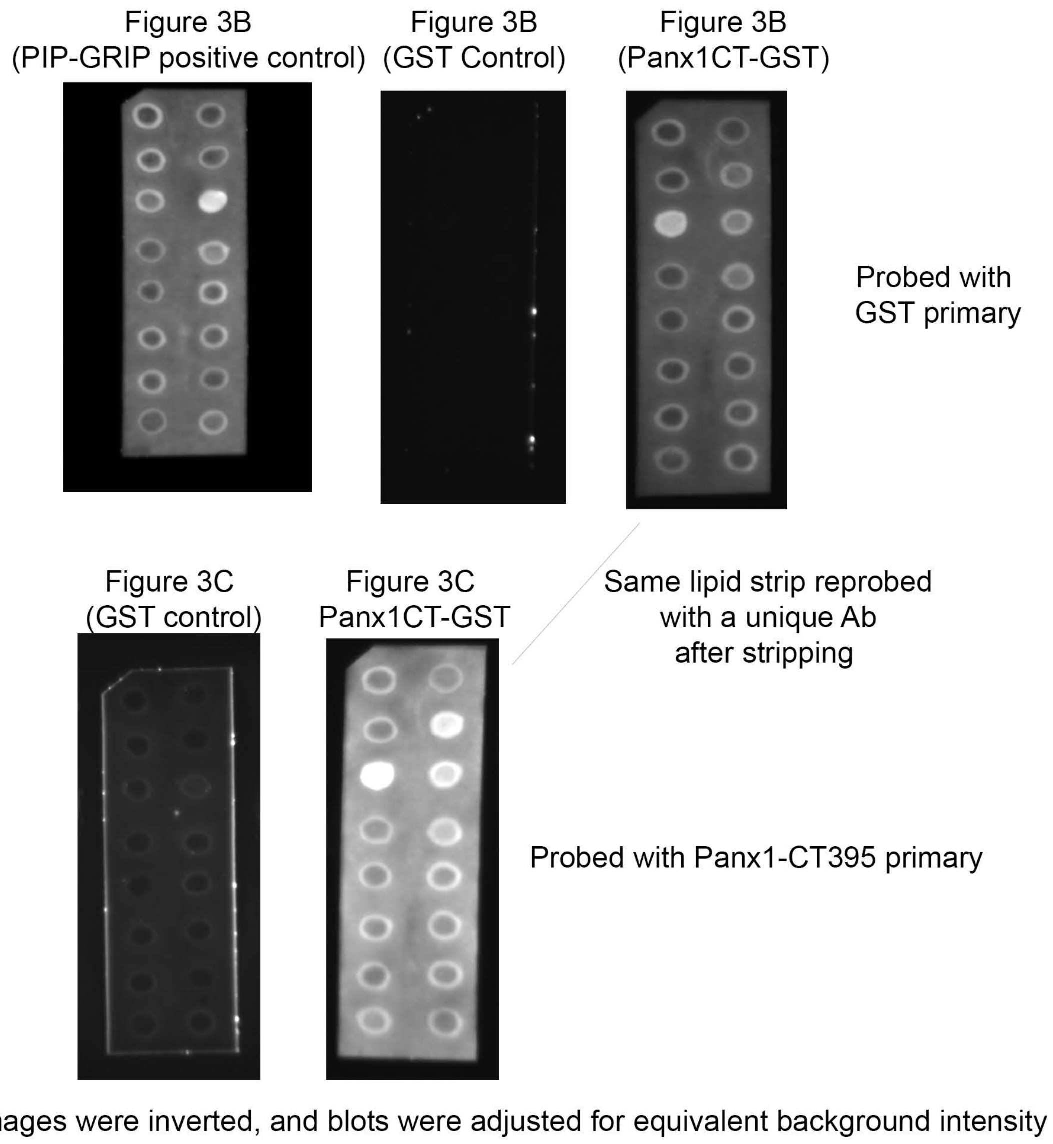

Fig. S2. Raw data and procedure for Fig. 3.

Biology Open • Supplementary information
